# *In vitro* differentiation of single donor derived human dental mesenchymal stem cells into pancreatic β cell-like cells

**DOI:** 10.1042/BSR20182051

**Published:** 2019-05-21

**Authors:** Sharath B. Shivakumar, Hyeon-Jeong Lee, Young-Bum Son, Dinesh Bharti, Sun A. Ock, Sung-Lim Lee, Young-Hoon Kang, Bong-Wook Park, Gyu-Jin Rho

**Affiliations:** 1 Department of Theriogenology and Biotechnology, College of Veterinary Medicine, Gyeongsang National University, Jinju 660-701, Republic of Korea; 2 Animal Biotechnology Division, National Institute of Animal Science, Rural Development Administration, Wanju-gun, Jeollabuk-do 565-851, Republic of Korea; 3 Department of Oral and Maxillofacial Surgery, Changwon Gyeongsang National University Hospital, Changwon 51472, Republic of Korea; 4 Research Institute of Life Sciences, Gyeongsang National University, Jinju 660-701, Republic of Korea

**Keywords:** MSCs, Pluripotency, Diabetes, Pancreatic lineage, Glucose challenge, Dithizone

## Abstract

The present study was carried out to investigate and compare the *in vitro* differentiation potential of mesenchymal stem cells (MSCs) isolated from human dental tissues (pulp, papilla, and follicle) of the same donor. MSCs were isolated from dental tissues (pulp, papilla, and follicle) following digestion method and were analyzed for the expression of pluripotent markers and cell surface markers. All three types of MSCs were evaluated for their potential to differentiate into mesenchymal lineages. Further, the MSCs were differentiated into pancreatic β cell-like cells using multistep protocol and characterized for the expression of pancreatic lineage specific markers. Functional properties of differentiated pancreatic β cell-like cells were assessed by dithizone staining and glucose challenge test. All three types of MSCs showed fibroblast-like morphology upon culture and expressed pluripotent, and mesenchymal cell surface markers. These MSCs were successfully differentiated into mesenchymal lineages and transdifferentiated into pancreatic β cell-like cells. Among them, dental follicle derived MSCs exhibits higher transdifferentiation potency toward pancreatic lineage as evaluated by the expression of pancreatic lineage specific markers both at mRNA and protein level, and secreted higher insulin upon glucose challenge. Additionally, follicle-derived MSCs showed higher dithizone staining upon differentiation. All three types of MSCs from a single donor possess similar cellular properties and can differentiate into pancreatic lineage. However, dental follicle derived MSCs showed higher potency toward pancreatic lineage than pulp and papilla derived MSCs, suggesting their potential application in future stem cell based therapy for the treatment of diabetes.

## Introduction

Diabetes mellitus (DM) is one of the devastating disease characterized either by absolute insulin deficiency due to the destruction of β cells (Type 1) or by relative insulin deficiency due to the reduced insulin sensitivity in peripheral cells (Type 2) [1]. DM results in abnormal plasma glucose level and the long-term hyperglycemia can cause severe complications such as diabetic nephropathy, diabetic retinopathy, cardiovascular disease, cataract, and neuropathy [2,3]. The exogenous insulin replacement is considered as a primary therapeutic approach to control plasma glucose levels. However, it does not match the precision of functioning β cells [4], and also it imposes psychological, physical, and financial burden on patients [5]. Even short- and long-acting, genetically synthesized insulin fails to match the sensitivity of nutrient induced endogenous insulin production, resulting in undesirable outcomes [3]. Transplantation of intact pancreas and/or pancreatic islets may be an ideal alternative. However, the shortage of cadaveric organs and the life-long dependency on immunosuppressive drugs limited this technique. Therefore, stem cell therapy is expected to be more powerful and an immediate alternative for this pervasive and debilitating disease. Indeed, the safety and efficacy of stem cell based therapy for the treatment of Type 1 and Type 2 diabetes has been reported in 2007 and 2008, respectively [6,7]. To date, pancreatic β cell-like cells were derived from various stem cell sources such as embryonic stem cells (ESCs) [8], induced pluripotent stem cells (iPSCs) [9], adipose tissue-derived mesenchymal stem cells (ADMSCs) [10], bone marrow-derived mesenchymal stromal cells (BMMSCs) [11], umbilical cord blood cells (UCBMSCs) [12], Wharton’s jelly mesenchymal stem cells (WJMSCs) [13], and dental tissue derived mesenchymal stem cells [14]. The use of ESCs for deriving pancreatic β cells is limited due to ethical considerations and practical issues such as the lack of available embryos, immunocompatability, and the risk of teratoma formation. Although with the technological advancement, iPSCs have been developed to derive patient specific β cells but the teratogenic potential of iPSCs remains major obstacle. In this context, mesenchymal stem cells (MSCs) have emerged as a promising autologous source of stem cells having capacity for self-replication, and multi-lineage differentiation potency to overcome the limitations of ethical issues, organ availability, and allogenic rejection.

Although MSCs have been isolated from various sources, the dental tissue derived MSCs are considered to be a suitable candidate for future regenerative applications owing to their ease of collection. Nevertheless, dental tissue is a routing biomedical waste from which potential MSCs can be isolated without any ethical issues. So far, MSCs have been isolated from different anatomical regions of dental tissue [15], which exhibits fibroblast like morphology, express cell surface, pluripotent markers, and differentiate into mesodermal lineages such as adipocytes, osteocytes, and chondrocytes [16]. Further, these cells possess transdifferentiation potency and they can successfully differentiate toward neuronal lineage [16], hepatocytes [17], and pancreatic cells [14]. It is considered that dental stem cells have a neural crest origin and have analogous properties to that of neural crest [18]. However, MSCs isolated from dental pulp tissue (DPSCs) have also been reported to express some endodermal markers such as GATA6, GATA4, and SOX17 [19]. This may be due to that fact that pancreatic β cells of endodermal origin share many common features with those of neurons of ectodermal origin [20]. Further, the sub-populations of neurons are found to express many of the essential functional elements associated with pancreatic β cells such as ion channels and glucose transporters [21], and pancreatic β cells also express neurotransmitter biosynthetic enzymes [22–24]. These intrinsic similarities between neural cells and DPSCs suggest that stem cells of dental origin may serve a suitable candidates for generating insulin-producing cells *in vitro*. However, there has been no report on comparative characterization of MSCs derived from different anatomical regions of dental tissue of single donor for their differentiation potential toward pancreatic insulin producing cells. Therefore, the present study was undertaken to determine and compare whether MSCs isolated from pulp, papilla, and follicle dental tissue could be differentiated into pancreatic β cell-like cells.

## Materials and methods

Unless otherwise specified, all chemicals and media used in the present study were purchased from Sigma (St. Louis, MO, U.S.A.) and Gibco (Life Technologies, U.S.A.), respectively.

### Isolation of MSCs from dental tissue and *in vitro* culture

MSCs were isolated from human dental pulp, papilla, and follicle tissues of a single tooth donor sample as previously described [25]. In brief, third molar were collected from male donors aged 14–18 years at the Department of Oral and Maxillofacial Surgery at Changwon Gyeongsang National University Hospital following approval by the Institutional Review Board of the University Hospital, and with the informed consent of enrolled patients for their tissue donation (GNUH IRB-2012-09-004). The dental pulp tissue was separated from the pulp changer of dental crown after fracture with bone forceps, dental follicle was separated from the tooth surface, and papilla was plucked from the apical part of the tooth by sung sterile scalpel. The tissue samples were rinsed with Dulbecco’s phosphate buffer saline (DPBS) containing 1% penicillin-streptomycin (10,000 IU and 10,000 μg/ml, respectively; Pen-Strep). The tissues were then chopped into pieces and digested in DPBS supplemented with 1 mg/ml collagenase type I at 37°C in an incubator with gentle agitation for 40 min. Following digestion, in order to obtain single cell suspension, the cell suspensions were filtered sequentially through a 100 and 40 μm nylon cell strainer (BD Falcon, Bedford, MA, U.S.A.) after preventing further digestion by adding Advanced Dulbecco’s modified Eagle’s media (ADMEM) supplemented with 10% fetal bovine serum (FBS). The cell suspensions were then centrifuged at 500 × *g* for 5 min, supernatants were discarded and the pellets were reconstituted in ADMEM supplemented with 10% FBS (10% ADMEM). The reconstituted cell suspensions were then seeded in 10 cm culture dishes containing 10% ADMEM and kept at 37°C in a humidified incubator containing 5% CO_2_ in air. Upon reaching 70–80% confluence, cells were dissociated with 0.25% (W/V) trypsin-EDTA solution and sub-cultured until passage 3. Cells from passage 3 were used for further characterization and analysis unless otherwise specified.

### Culture of INS-1 rat insulinoma cells

INS-1 rat insulinoma cells were cultured in RPMI 1640 medium supplemented with 10% FBS containing 1% penicillin-streptomycin (10,000 IU and 10,000 μg/ml, respectively; Pen-Strep) and maintained at 37°C in a humidified incubator containing 5% CO_2_ in air.

### Morphology of cultured MSCs and INS-1 rat insulinoma cells

Morphology of *in vitro* cultured MSCs and INS-1 rat insulinoma cells was analyzed under a light microscope in all the experiments. Images were taken at 100× magnification using Nikon DIAPHOT 300, Japan.

### Evaluation of cell proliferation

All three types of MSCs were evaluated for their proliferation ability by using MTT [3-(4,5-dimethylthiazol-2yl)-2, 5-diphenyltetrazolium bromide] assay. In brief, cells were seeded at a density of 9 × 10^3^ cells/well on 24-well plate and cultured in 10% ADMEM medium. The MTT assay was performed in triplicates in three independent experiments. After culturing for specified time of interval (24, 48, 72 and 96 h), MTT (Sigma) was added to each well at a final concentration of 1 mg/ml and incubated at 37°C for 4 h. After removing media, cells were washed twice with DPBS. The insoluble formazan, a product formed when MTT is metabolized by viable cells was dissolved with dimethyl sulphoxide (DMSO; Sigma) and the colored product formed was collected and the absorbance was measured at 570 nm using a plate reader.

### Phenotyping and cell cycle analysis

MSCs derived from human dental pulp, papilla, and follicle tissues were analyzed for cell surface marker’’s (Cluster differentiation; CD) expression using flow cytometer (BD FACSVerse, Becton Dickinson, NJ, U.S.A.) as previously described [26]. In brief, cells were fixed with 3.7% formaldehyde for 30 min after harvesting 80% confluent cells. Then cells were washed twice with DPBS and incubated with fluorescence isothiocyanate (FITC) conjugated CD34 (1:100, BD Pharmingen, CA, FITC Mouse Anti-Human CD34), CD45 (1:100, BD Pharmingen, FITC Mouse Anti-Human CD45), CD90 (1:100, BD Pharmingen, FITC Mouse Anti-Human CD90), CD73 (1:100, BD Pharmingen, FITC Mouse Anti-Human CD73), and unconjugated CD14 (1:100, Santa Cruz Biotechnology, Mouse Anti-Human CD14), CD19 (1:100, Santa Cruz Biotechnology, Mouse Anti-Human CD19), HLA-DR (1:100, Santa Cruz Biotechnology, Mouse Anti-HLA-DR), CD105 (1:100, Santa Cruz Biotechnology, Mouse Anti-Human CD105), and intracellular marker vimentin (1:100, Sigma-Aldrich, Mouse Anti-Human vimentin) for 30 min under dark. Unconjugated primary antibodies were then treated with secondary FITC-conjugated goat anti-mouse IgG (1:100, BD Pharmingen) for 30 min under dark. Mouse IgG1 (1:100, BD Pharmingen) was used as isotype matched negative control. A total of 10,000 labeled cells were acquired and analyzed for each sample.

For cell cycle analysis, a total of 1 × 10^6^ cells per ml were fixed in 70% ethanol at 4°C for 4 h. Cells were then washed twice with DPBS and stained with 10 μg/ml propidium iodide (PI) solution for 15 min. The DNA content was measured and categorized as G_0_/G_1_, S, or G_2_/M phase of the cell cycle.

### *In vitro* differentiation into mesenchymal lineages

Human dental pulp, papilla, and follicle tissues derived MSCs were evaluated for their differentiation potency toward mesenchymal lineages by following previously published protocol [27]. In brief, all three types of MSCs were cultured in 10% ADMEM supplemented with lineage-specific constituents for a period of 21 days by changing the media for every 3 days interval. Osteogenesis was induced with media containing 50 μM ascorbate-2-phosphate, 0.1 μM dexamethasone, and 10 mM glycerol-2-phosphate, and was confirmed by the accumulation of calcium nodules by staining with alizarin red and von Kossa. Adipogenesis was induced with media containing 10 μM insulin, 1 μM dexamethasone, 100 μM indomethacin, and 500 μM isobutylmethylxanthine (IBMX), and was confirmed by the formation of lipid droplets by staining with Oil red O solution. Chondrogenesis was induced with commercial chondrogenic medium (StemPro® Osteocyte/Chondrocyte Differentiation Basal Medium; StemPro® Chondrogenesis supplement, Gibco by life technology), and was confirmed by the deposition of glycosaminoglycans and proteoglycans by staining with Safranin O and Alcian blue respectively.

### *In vitro* differentiation into pancreatic β cell-like cells

MSCs derived from human dental pulp, papilla, and follicle tissues were differentiated into insulin producing pancreatic β cell-like cells as described previously with minor modifications [28]. In brief, the differentiation was carried out in four different steps:

Step I: After reaching 70% confluence, cells were cultured in DMEM containing 10% FBS and 25 mM glucose supplemented with 1 μM retinoic acid for 24 h.

Step II: After induction, cells were cultured for another 2 days in DMEM containing 10% FBS and 25 mM glucose, then dissociated with 0.25% trypsin-EDTA solution.

Step III: Dissociated cells were plated on to a six-well plate precoated with Geltrex LDEV-free membrane matrix and cultured in DMEM containing 10% FBS and 5.6 mM glucose, supplemented with 10 mM nicotinamide, 10 ng/ml epidermal growth factor (EGF) and 300 nM indolactam V for 9 days. The media were changed for every 3 days interval during differentiation.

Step IV: The media were changed to DMEM containing 10% FBS and 5.6 mM glucose, supplemented with 10 mM Exendin-4 and 50 ng/ml Activin A for 7 days. The media were changed for every 3 days interval during differentiation.

Control cells were maintained in parallel using DMEM containing 10% FBS and 25 mM glucose.

### Glucose-stimulated insulin secretion (GSIS)

Upon pancreatic differentiation of MSCs, both control and differentiated cells were analyzed for their ability to secrete insulin upon glucose challenge following previous protocol with minor modifications [28,29]. In brief, cells were washed thrice with Krebs (Krb) buffer and were then preincubated in low glucose (2 mM) Krb for 2 h in order to remove the residual insulin. Cells were then washed again with Krb and incubated in low glucose Krb for 30 min, and the supernatant was collected. After washing cells with Krb, incubated in high glucose (20 mM) Krb for 30 min, and the supernatant was collected. Finally, cells were washed with Krb and incubated in Krb buffer containing 2 mM glucose and 30 mM KCl for depolarization challenge for a period of 30 min, and the supernatant was collected. The cell numbers were noted using hemocytometer upon trypsinization and the insulin content in the supernatant was estimated using Human INS ELISA Kit (neo SCIENTIFIC).

### Dithizone (DTZ) staining

Upon pancreatic differentiation of MSCs, both control and differentiated cells were analyzed for the presence of intracellular zinc by staining with dithizone. In brief, a stock solution of dithizone was prepared by dissolving 100 mg of dithizone in 5 ml of DMSO. Both control and differentiated cells were washed twice with DPBS and stained with 10 μl dithizone stock in 1 ml DPBS solution at 37°C for 15 min. The stained cells were examined under a phase-contrast microscope (Nikon) for crimson-red staining and images were taken.

### Polymerase chain reaction (PCR) and real-time quantitative polymerase chain reaction (RT-qPCR) analysis

The expression of pluripotent marker genes was analyzed by PCR, and the fold change in the expression of mesenchymal lineage specific genes and pancreatic lineage specific genes was evaluated by RT-qPCR in triplicates from three independent experiments. Total RNA was isolated using easy-spin™ [DNA free] total RNA extraction kit (iNtRON Biotechnology). A total of 2 μg RNA was used to synthesize complementary DNA (cDNA) using HiSenScript™ RH[-] RT PreMix Kit (iNtRON Biotechnology) following reaction in three steps; step I: 42°C for 50 min, step II: 85°C for 10 min, step III: End. The PCR reaction containing 2 μl cDNA, 1 μl each of forward and reverse primers of 10 μM concentration and 16 μl of RNase free water was carried out using Maxime PCR Premix (iNtRON Biotechnology). The PCR reaction was carried out in following conditions: initial denaturation at 94°C for 2 min, followed by 35 cycles of denaturation at 94°C for 30 s, annealing at 55–60°C for 20 s, extension at 72°C for 30 s and final extension at 72°C for 10 min, using Mastercycler® pro (Eppendorf, Germany). The PCR products were analyzed by agarose gel electrophoresis.

The Real-time qPCR was carried out in Rotor gene Q (Qiagen) using RealMOD™ Green AP 5× qPCR mix (iNtRON Biotechnology). Each reaction mix with a final volume of 25 μl consisted of a total of 50 ng cDNA, 5 μl qPCR mix, 13 μl RNase free water, and 1 μl each of forward and reverse primers at 400 nM final concentration. The reaction was carried out with an initial denaturation at 95°C for 10 min, followed by 40 PCR cycles of 95°C for 10 s, annealing (Primer specific temperature) for 6 s, and 72°C for 4 s, followed by a melting curve from 60°C to 95°C at 1°C/s and then cooling at 40°C for 30 s, according to manufacturer’s instruction. The threshold cycle (CT) values and melting curves of each sample were analyzed using Rotor-Gene Q series software (Qiagen). YWHAZ (Tyrosine 3-monooxygenase/tryptophan 5-monooxygenase activation protein, zeta polypeptide) was used as housekeeping gene for normalization of the data. The relative mRNA abundance was calculated according to the 2^−∆∆CT^ method. The specificity of primers was analyzed by their product length on agarose gel. The primers used in the present study are listed in Table 1.

**Table 1:** List of primers used in the study

Gene	Primer sequence	Product size (bp)	Annealing temp (°C)	Accession no.
*OCT4*	F: AAGCAGCGACTATGCACAAC	140	59	NM_002701.5
	R: AGTACAGTGCAGTGAAGTGAGG			
*SOX2*	F: CACCCACAGCAAATGACAGC	120	59	NM_003106.3
	R: AGTCCCCCAAAAAGAAGTCCAG			
*NANOG*	F: GCAGATGCAAGAACTCTCCAAC	133	55	AB093576.1
	R: CTGCGTCACACCATTGCTATTC			
*PPARγ*	F: TTGCTGTCATTATTCTCAGT	124	60	AB565476.1
	R: GAGGACTCAGGGTGGTTCAG			
*FABP4*	F: TGAGATTTCCTTCATACTGG	128	60	NM_001442.2
	R: TGGTTGATTTTCCATCCCAT			
*LPL*	F: AGACACAGCTGAGGACACTT	137	60	NM_000237.2
	R: GCACCCAACTCTCATACATT			
*RUNX2*	F: ATGTGTGTTTGTTTCAGCAG	199	60	NM_001024630.3
	R: TCCCTAAAGTCACTCGGTAT			
*OSTEONECTIN*	F: GTGCAGAGGAAACCGAAGAG	202	60	J03040.1
	R: AAGTGGCAGGAAGAGTCGAA			
*BMP2*	F: TAGACCTGTATCGCAGGCAC	149	60	NM_001200.2
	R: GGTTGTTTTCCCACTCGTTT			
*SOX9*	F: ATGGAGCAGCGAAATCAACG	118	60	BC007951.2
	R: CAAAGTCCAAACAGGCAGAGAG			
*AGGRECAN*	F: GAATGGGAACCAGCCTATACC	98	60	NM_001135.3
	R: TCTGTACTTTCCTCTGTTGCTG			
*COLLAGEN II*	F: GAGACCTGAAACTCTGCCACC	165	60	NM_001844.4
	R: TGCTCCACCAGTTCTTCTTGG			
*PDX1*	F: GCTGGCTGTCATGTTGAACTTG	59	60	NM_000209.3
	R: GGCGGTTTTGGAACCAGAT			
*NKX6.1*	F: AGAGAGTCAGGTCAAGGTCTGGTT	103	60	NM_006168.2
	R: TGTCTCCGAGTCCTGCTTCTTCTT			
*NGN3*	F: GAAGTGGGCATTGCAAAGTG	94	60	NM_020999.3
	R: GGGTAGTGCTACCATTCTAGTATTC			
*ARX*	F: CACTCAGCGTGGTATGGTAAA	76	55	NM_139058.2
	R: GTCTAGGAACCCTACCGTATCT			
*PAX4*	F: TGGGAAGGAGATGGCATAGA	98	55	NM_006193.2
	R: ATCACAGGAAGGAGGAAGGA			
*INSULIN*	F: GCAGCCTTTGTGAACCAACAC	67	55	JF909299.1
	R: CCCCGCACACTAGGTAGAGA			
*GLUT2*	F: CCTAGGCAGAGCTGCGAATAA	61	55	NM_000340.1
	R: GTGTGAGTGTGGCACATGCA			
*MAFA*	F: TGAGGTTGAGCGGAGAA	108	57	NM_201589.3
	R: AAGGTGGGAACGGAGAA			
*GLUCAGON*	F: AAGCATTTACTTTGTGGCTGGATT	91	57	NM_002054.4
	R: TGATCTGGATTTCTCCTCTGTGTCT			
*SOMATOSTATIN*	F: TGTCTGAACCCAACCAGA	90	57	NM_001048.3
	R: GCAGCTCAAGCCTCATTT			
*YWHAZ*	F: CTTCACAAGCAGAGAGCAAAG	102	55	NM_003406.3
	R: CGACAATCCCTTTCTTGTCATC			

### Immunocytochemical analysis

The expression of pancreatic lineage specific marker proteins was evaluated by immunocytochemistry. In brief, the differentiated pancreatic β cell-like cells and INS-1 rat insulinoma cells were fixed with 3.7% formaldehyde for 30 min and permeabilized with 0.25% Triton X-100 for 10 min at room temperature. After thorough washing with DPBS, cells were blocked with 1% BSA for 1 h and incubated with primary antibodies such as rabbit anti-PDX1 (1:200, Cell Signaling Technology), rabbit anti-NGN3 (1:200, abcam), mouse anti-Arx (1:200, Santa Cruz Biotechnology), rabbit anti-PAX4 (1:200, Santa Cruz Biotechnology), mouse anti-MafA (1:200, Santa Cruz Biotechnology), rabbit anti-Glut2 (1:200, abcam), mouse anti-C peptide (1:200, abcam) or mouse anti rat C peptide (1: 500, BioRad), mouse anti-Glucagon (1:200, abcam), goat anti-Somatostatin (1:200, Santa Cruz Biotechnology), and mouse anti-Insulin (1:200, Santa Cruz Biotechnology) or mouse anti-Insulin (1:500, Invitrogen) for overnight at 4°C. Cells were then washed twice with DPBS and incubated with CruzFluor 488 conjugated goat anti-rabbit IgG (1:200, Santa Cruz Biotechnology), or CruzFluor 488 conjugated donkey anti-goat IgG (1:200, Santa Cruz Biotechnology), or CruzFluor 594 conjugated donkey anti-rabbit IgG (1:200, Santa Cruz Biotechnology), or CruzFluor 594 conjugated donkey anti-mouse IgG (1:200, Santa Cruz Biotechnology), or FITC conjugated donkey anti-mouse IgG (1:200, Santa Cruz Biotechnology) for 1 h at 37°C. The nuclei were stained with DAPI at a concentration of 1 μg/ml for 5 min and the images were captured using fluorescence microscope (Leica, Germany).

### Western blotting analysis

The expression of pluripotent marker proteins in MSCs derived from dental pulp, papilla, and follicle tissues was evaluated by Western blotting. In brief, the lysates of protein were prepared from cultured MSCs using RIPA buffer (Thermo Scientific, Rockford, IL, U.S.A.) containing protease inhibitors. The concentration of protein was measured using Microplate BCA Protein Assay kit (Pierce Biotechnology, Rockford, IL, U.S.A.) and a total of 25 μg protein was separated by 12% sodium dodecyl sulfate-polyacrylamide gel electrophoresis (SDS-PAGE, Mini Protean, BioRad, Hercules, CA, U.S.A.). The separated proteins were then transferred onto polyvinylidene difluoride membranes (PVDF, Millipore) and membranes were incubated with primary antibodies such as rabbit anti-OCT4 (1:200, abcam), rabbit anti-SOX2 (1:200, abcam), rabbit anti-NANOG (1:200, abcam), and mouse anti-GAPDH (1:200, Millipore) for overnight at 4°C. Then membranes were incubated with horseradish peroxidase (HRP)-conjugated goat anti-rabbit IgG (1:10,000, Santa Cruz Biotechnology), and goat anti-mouse IgG (1:10,000, Santa Cruz Biotechnology) secondary antibodies for 2 h at room temperature. The immunoreactivity was detected in dark condition by adding enhanced chemiluminescence (ECL; Supersignal, West Pico Chemiluminescent substrate, PIERCE, IL) and exposed to X-ray films.

### Statistical analysis

The statistical differences between experiments groups were analyzed by one-way ANOVA using IBM SPSS 21.0. Tukey’s test was performed for multiple comparisons and data were presented as a mean ± standard error of the estimate of mean value (S.E.M) for each sample analyzed in triplicates obtained from three independent experiments. The value *P*<0.05 is considered as significant difference.

## Results

### Morphology, proliferation, phenotyping, and expression of pluripotent markers

**Figure 1:**
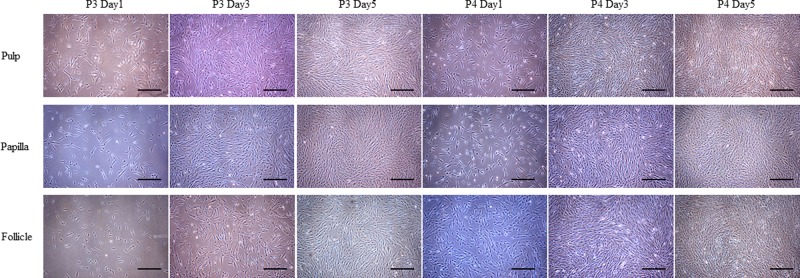
Phase contrast microscopic images MSCs isolated from dental tissues such as pulp, papilla, and follicle showed adherent fibroblast-like morphology at different time intervals at passages P3 and P4; scale bar = 100 μm.

**Figure 2:**
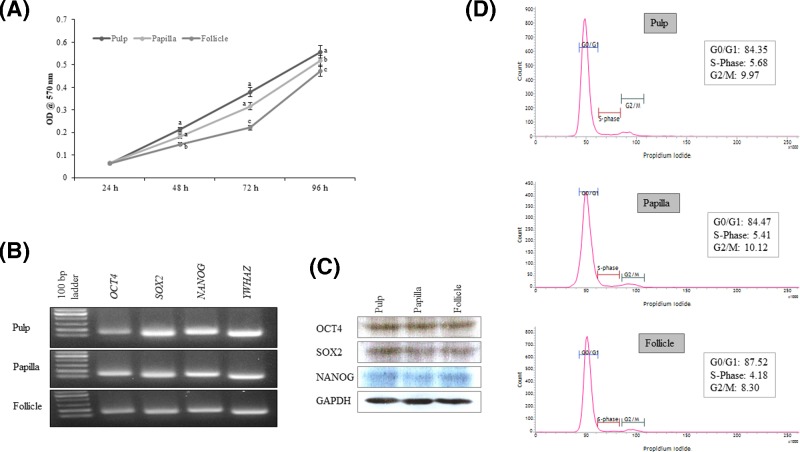
Characterization of MSCs isolated from dental tissues such as pulp, papilla, and follicle (**A**) Analysis of cell proliferation using MTT assay. Data are represented for each sample conducted in triplicates from three independent experiments. Significant differences are indicated using different letters when *P*<0.05. (**B**) Analysis of the expression of pluripotent marker genes using polymerase chain reaction that was evaluated by agarose gel electrophoresis. All three types of MSCs expressed octamer-binding transcription factor 4 (*OCT4*), sex determining region Y-box 2 (*SOX2*), and *NANOG*. (**C**) Western blot images showing the expression of pluripotent marker proteins such as OCT4, SOX2, and NANOG in MSCs isolated from pulp, papilla, and follicle dental tissues. (**D**) Flow cytometric analysis of three types of MSCs at passage 3 demonstrating normal DNA content in gap0/1 (G0/G1), synthesis (S), or gap2/mitotic (G2/M) phases of the cell cycle. A total of 10,000 MSCs were counted for each sample in triplicates from three independent experiments.

**Figure 3:**
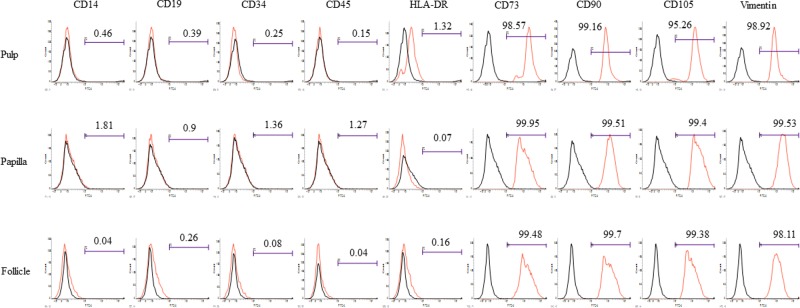
Flow cytometric analysis of the expression of cell surface markers in MSCs isolated from pulp, papilla, and follicle dental tissues All three types of MSCs were positive for the expression of mesenchymal markers such as CD73, CD90, CD105, and internal marker vimentin, whereas these cells were negative for the expression of monocyte marker CD14, B-lymphocyte marker CD19, HLA-DR, and hematopoietic markers CD34 and CD45.

The MSCs isolated from human dental pulp, papilla, and follicle tissues exhibited adherent fibroblast like spindle morphology that become homogeneous at passage 3 upon sub culturing (Figure 1). The pulp derived MSCs had higher proliferation rate compared with papilla and follicle derived MSCs as assessed by MTT assay (Figure 2A). However, the flow cytometric analysis of cell cycle revealed no significant differences among three types of MSCs (Figure 2D). The pluripotent markers such as OCT4, SOX2, and NANOG were positively expressed in all three types of MSCs as evaluated by PCR and Western blotting (Figure 2B,C). The expression of cell surface markers was analyzed by flow cytometer. All MSCs were positive for the expression of CD73, CD90, CD105, and vimentin, and were negative for the expression of CD14, CD19, CD34, CD45, and HLA-DR (Figure 3).

### *In vitro* mesenchymal lineage differentiation capacity

**Figure 4:**
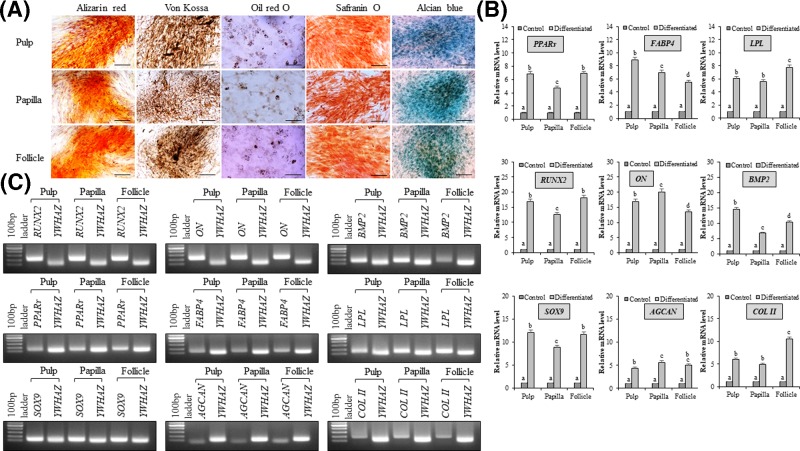
Analysis of *in vitro* mesenchymal lineage differentiation (**A**) MSCs isolated from dental pulp, papilla, and follicle tissues were differentiated into mesenchymal lineages such as osteocytes, adipocytes, and chondrocytes. The differentiation was confirmed by lineage-specific staining (osteocytes: alizarin red and von Kossa; adipocytes: oil red O; chondrocytes: safranin O and alcian blue); scale bar = 100 μm. (**B**) RT-qPCR analysis of mRNA expression of mesenchymal lineage specific marker genes. The relative mRNA level was quantified using 2^−∆∆CT^ method using tyrosine 3-monooxygenase/tryptophan 5-monooxygenase activation protein, zeta polypeptide (*YWHAZ*) for normalization. The mRNA levels are expressed as fold change in relation to the undifferentiated control MSCs [peroxisome proliferator-activated receptor *γ*-2 (*PPARγ*), fatty-acid binding protein 4 (*FABP4*), and lipoprotein lipase (*LPL*), adipocyte-specific; runt-related transcription factor 2 (*RUNX2*), osteonectin (*ON*), and bone morphogenetic protein 2 (*BMP2*), osteocyte-specific; and sex determining region Y-box 9 (*SOX9*), cartilage-specific proteoglycan core protein (*AGGRECAN*), and type II collagen (*COLLAGEN II*), chondrocyte-specific]. Data represent the mean ± SE obtained in triplicates from three independent experiments. Significant differences are shown using different letters when *P*<0.05. (**C**) The product size of each marker genes was evaluated by agarose gel electrophoresis.

MSCs isolated from human dental pulp, papilla, and follicle tissues were successfully differentiated into osteocytes, adipocytes, and chondrocytes upon lineage specific induction. The formation of mineralized nodules and calcium deposits demonstrated by staining with alizarin red and von Kossa confirmed osteogenesis (Figure 4A). Further, the RT-qPCR analysis of the expression of lineage specific marker genes such as runt-related transcription factor-2 (*RUNX2*), osteonectin (*ON**)***, and bone morphogenetic protein 2 (*BMP2*) showed significant (*P*<0.05) increase when compared with undifferentiated controls (Figure 4B,C). The accumulation of intracellular lipid droplets in adipocyte differentiated cells as demonstrated by Oil red O staining confirmed adipogenesis (Figure 4A), and the expression of adipogenic lineage specific marker genes such as peroxisome proliferative activated receptor *γ* (*PPARγ*), fatty acid binding protein 4 (*FABP4*), and lipoprotein lipase (*LPL*) was found to be significantly (*P*<0.05) increased when compared with undifferentiated controls (Figure 4B,C). Further, the deposition of glycosaminoglycans and proteoglycans in differentiated cells as demonstrated by staining with Safranin O and Alcian blue confirmed the chondrogenesis (Figure 4A), and the expression of chondrogenic lineage specific marker genes such as SRY-Box 9 (*SOX9*), aggrecan (*AGCAN*), and collagen II (*COL II*) was found to be significantly (*P*<0.05) increased when compared with undifferentiated controls (Figure 4B,C).

### *In vitro* pancreatic β cell-like cells differentiation capacity of dental pulp, papilla, and follicle tissue derived MSCs

**Figure 5:**
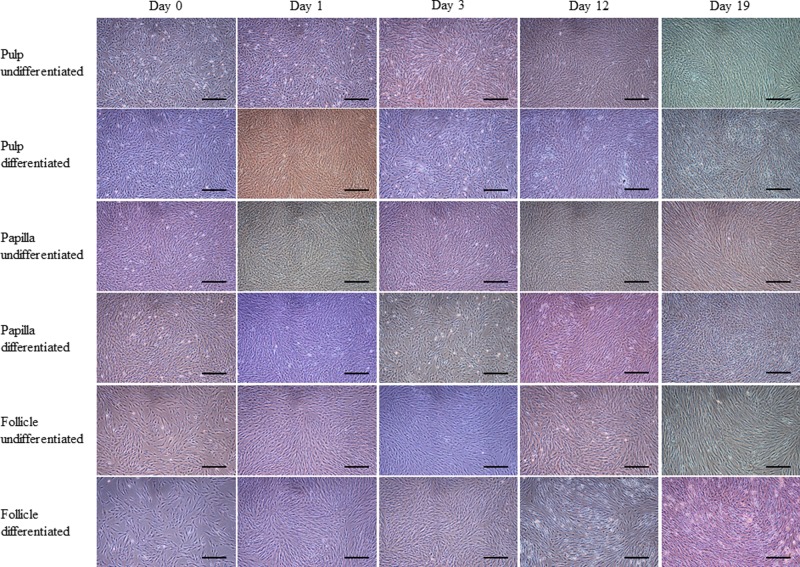
Phase contrast images showing morphological changes occurred during *in vitro* differentiation of MSCs isolated from dental pulp, papilla, and follicle tissues into pancreatic β cell-like cells All three types of MSCs were successfully differentiated toward pancreatic lineage and showed change in morphologies at different stages of differentiation; scale bar = 100 μm.

**Figure 6:**
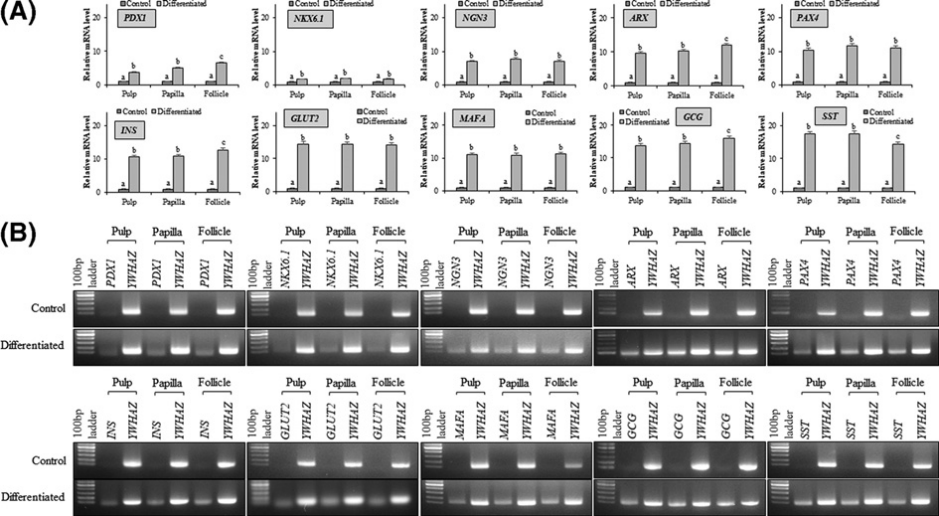
Analysis of the expression of pancreatic lineage specific marker genes after differentiation (**A**) RT-qPCR analysis for the evaluation of fold change in the expression of pancreatic lineage specific marker genes such as pancreatic duodenal homeobox-1 (*PDX-1*), NK6 homeobox 1 (*NKX6.1*), neurogenin 3 (*NGN3*), aristaless-related homeobox (*ARX*), paired box 4 (*PAX4*), insulin (*INS*), solute carrier family 2 member 2 (*GLUT2*), MAF bZIP transcription factor A (*MAFA*), glucagon (*GCG*), and somatostatin (*SST*). The expression levels are expressed as change in relative mRNA level in relation to the undifferentiated control using 2^−∆∆CT^ method. Tyrosine 3-monooxygenase/tryptophan 5-monoxygenase activation protein, zeta polypeptide (*YWHAZ*) was used for data normalization. The data are represented as mean ± SE obtained in triplicates from three independent experiments. Significant differences are denoted using different letters when *P*<0.05. (**B**) The product size of each marker genes was evaluated by agarose gel electrophoresis.

**Figure 7:**
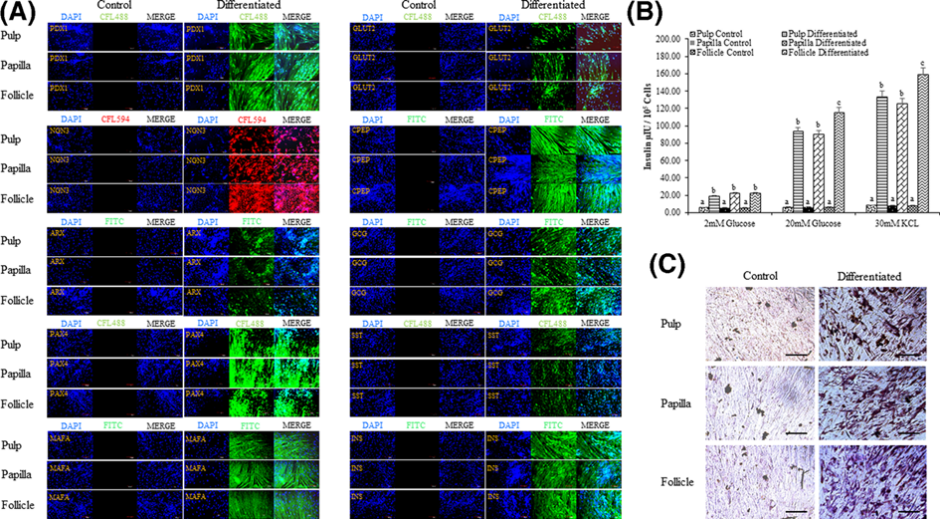
Immunocytochemistry and functional evaluation of differentiated pancreatic β cell-like cells (**A**) Analysis of the expression of pancreatic lineage specific marker proteins by immunocytochemistry; scale bar = 100 μm. (**B**) Evaluation of insulin secretion by glucose challenge test. The data are represented as mean ± SE obtained in triplicates from three independent experiments. Significant differences are denoted using different letters when *P*<0.05. (**C**) Differentiated cells were evaluated for the presence of zinc ions by staining with dithizone; scale bar = 100 μm.

In order to evaluate and compare the differentiation potency of MSCs from three different anatomical regions of dental tissue (pulp, papilla, and follicle), cells from passage 3 were induced to pancreatic lineage. After 19 days of induction, change in morphology from fibroblastic to spherical was observed and cells tended to form clumps when compared with control undifferentiated counterparts (Figure 5), though these changes were not similar to INS-1 rat insulinoma cells (Supplementary Figure S1A). However, no significant changes in morphology between all three types of MSCs were observed. Differentiated cells expressed pancreatic lineage specific marker genes as evaluated by RT-qPCR. The mRNA levels of pancreatic duodenal homeobox-1 (*PDX1*), NK6 homeobox 1 (*NKX6.1*), neurogenin 3 (*NGN3*), aristaless-related homeobox (*ARX*), paired box 4 (*PAX4*), insulin (*INS*), solute carrier family 2 member 2 (*GLUT2*), MAF bZIP transcription factor A (*MAFA*), glucagon *(GCG)*, and somatostatin (*SST*) were found to be significantly (*P*<0.05) increased after differentiation when compared with control undifferentiated cells, and the relative fold change in expression levels was 3.75 ± 1.6, 4.96 ± 1.2, 6.53 ± 2.0 and 1.69 ± 1.6, 1.9 ± 2.4, 1.86 ± 2.6 and 7.0 ± 2.8, 7.68 ± 1.4, 7.05 ± 2.2 and 9.66 ± 2.0, 10.21 ± 2.4, 11.91 ± 2.0 and 10.4 ± 2.2, 11.71 ± 2.6, 11.08 ± 1.2 and 10.65 ± 1.6, 10.83 ± 1.8, 12.59 ± 2.0 and 14.4 ± 1.8, 14.23 ± 2.8, 14.15 ± 2.0 and 10.97 ± 3.0, 10.91 ± 2.8, 11.18 ± 1.6 and 13.63 ± 2.4, 14.36 ± 2.0, 15.92 ± 3.4 and 17.41 ± 1.8, 17.5 ± 2.0, 14.37 ± 2.6, respectively, in pulp, papilla, and follicle tissues derived differentiated MSCs (Figure 6A,B). Further, immunocytochemical analysis of differentiated pancreatic β cell-like cells demonstrated the expression of pancreatic lineage specific marker proteins in all three types of MSCs (Figure 7A). The expression of C peptide and insulin proteins in pulp, papilla, and follicle tissues derived differentiated MSCs was comparable to that in INS-1 rat insulinoma cells as revealed by immunocytochemical analysis (Figure 7A and Supplementary Figure 1B). The analysis of glucose-stimulated insulin secretion (GSIS) demonstrated that MSCs derived from pulp, papilla, and follicle tissues have the ability to differentiate toward insulin secreting pancreatic β cell-like cells. The amount of insulin secreted in response to low glucose (2 mM glucose) was found to be 5.62 ± 2.4, 19.12 ± 4.2, 5.66 ± 3.4, 22.42 ± 3.4, 5.40 ± 3.0, and 22.37 ± 3.4 μIU/10^5^ cells, and which is further increased to 6.24 ± 2.8, 93.37 ± 1.8, 6.32 ± 2.8, 90.15 ± 2.4, 6.12 ± 3.4, and 115.29 ± 2.2 μIU/10^5^ cells in control pulp MSCs, differentiated pulp MSCs, control papilla MSCs, differentiated papilla MSCs, control follicle MSCs, and differentiated follicle MSCs, respectively upon high glucose (20 mM glucose) treatment (Figure 7B). Follicle-derived MSCs showed higher glucose sensitivity and secreted significantly (*P*<0.05) higher insulin upon glucose challenge when compared with pulp, and papilla-derived MSCs. Nevertheless, differentiated cells were positively stained with dithizone cytochemical solution (Figure 7C), demonstrating that MSCs found in dental tissue can successfully differentiate into pancreatic β-like cells possessing zinc content. Overall, the present study revealed that follicle-derived MSCs possess higher differentiation potency toward pancreatic lineage and can be successfully differentiated *in vitro* to insulin producing cells.

## Discussion

The present study was conducted to compare the multipotency of three types of dental MSCs isolated from a single donor tooth. MSCs isolated form dental pulp, papilla, and follicle tissues were compared on the basis of their morphology, proliferation, expression of cell surface markers and pluripotent transcription factors, *in vitro* differentiation into mesenchymal lineages and pancreatic lineage.

As dental tissue is considered a biomedical waste in routine dental procedures, it is gaining more attention in the field of regenerative medicine as a potential source of MSCs without imposing any ethical issues. Many dental-derived MSCs have been identified and characterized, including MSCs from human exfoliated deciduous teeth (SHED) [30–32], dental pulp [16,17], papilla [16], and follicle [16]. However, the properties of these MSCs present in different anatomical regions of dental tissue and their feasibility for application in future regenerative medicine still need to be investigated in greater detail. Thus, in the present study, we compared the multilineage potential of three different types of MSCs obtained from single tooth donor.

All three types of MSCs showed plastic adherent fibroblast-like morphology when cultured under identical conditions. MSCs of dental tissue possess more clonogenic as well as proliferative potentials than BMSCs [18,33]. Our study indicates that pulp-derived MSCs had higher proliferation rate than papilla, and follicle-derived MSCs. The phenotyping of three types of MSCs demonstrated the MSC-like phenotypic features and our results are in accordance with the previous results [18,25,33,34]. MSCs from dental pulp, papilla, and follicle tissues expressed key pluripotent transcription factors such as OCT4, SOX2, and NANOG invariably both at mRNA and protein levels. These pluripotent markers play a major role in self-renewability, pluripotency, and differentiation of embryonic stem cells [35] as well as multipotent MSCs irrespective of sources including dental tissue [30,33,36]. Therefore, our findings indicate the occurrence of highly primitive cells in dental pulp, papilla, and follicle tissues with potent stemness.

Present study also demonstrated that MSCs from dental pulp, papilla, and follicle tissues were successfully differentiated into mesenchymal lineages such as osteocytes, adipocytes, and chondrocytes under appropriate culture conditions. Differentiated cells also expressed lineage specific markers and positively stained for lineage specific staining. Pulp and follicle tissue derived MSCs exhibited higher differentiation potential toward mesenchymal lineages when compared with papilla-derived MSCs and these findings are consistent with previous report [37]. The previous studies failed to show the adipogenic potential of dental MSCs [18,38]; however, our study indicates the successful differentiation of all three types of MSCs to adipocyte lineage and this could be due to the supplementation of IBMX in our adipogenic induction medium. It has been reported that IBMX plays potent synergetic role in MSCs to undergo adipogenesis [39]. Therefore, it is reasonable to speculate that MSCs from dental pulp, papilla, and follicle tissues are slightly distinct, and possess different potential toward mesodermal lineages despite their identical donor origin.

We further assessed the transdifferentiation potential of dental pulp, papilla, and follicle tissues derived MSCs toward pancreatic β cell-like cells. Previous studies have demonstrated the ability of various human stem cells including dental MSCs to differentiate into pancreatic lineages with the use of different cytokines, growth factors, and extracellular matrix components [5,6,8–10,12–14]. However, the comparative analysis of transdifferentiation potency of different region derived dental MSCs to pancreatic lineage has not yet been reported. The selection of supplements and induction time of pancreatic lineage differentiation were based on previous report [28], and our protocol does not employ gene transfection and/or genetic modification rather rely on defined culture conditions. We have used Geltrex LDEV-free reduced growth factor basement membrane matrix for differentiation. Geltrex is a compound matrix consisting of different proteins secreted by EHS sarcoma cells including laminins, entactin, and collagens [40]. Re-plating the dissociated cells on a fresh extracellular matrix (ECM) may have beneficial effects on further differentiation into pancreatic lineage. ECM plays a crucial roles in directing stem cells toward a specific fate [41];, particularly, matrigel was shown to have a pro-endocrine effects based on the observation that pancreatic ductal epithelial cells up-regulated endocrine markers on an overlay with matrigel more than other matrices such as collagen [42]. Earlier, several studies have also reported the influence of components present in ECM on pancreatic development [43–45]. Retinoic acid signaling is an activator for PDX1 in the pancreas [46]; especially, it is involved in the generation of PDX1 expressing pancreatic progenitors in the zebrafish [47]. During Step III of our protocol, we used nicotinamide, EGF, and indolactam V. Nicotinamide is a poly adenosine diphosphate (ADP)-ribose synthetase inhibitor that can induce islet formation from pancreatic progenitor cells, differentiation and maturation of liver stem cells into insulin-producing cells, and also human fetal pancreatic islet cells [48–50]. The use of EGF can increase the number of undifferentiated endocrine progenitor cells, and the removal of EGF induces β-cell formation [51]. Another small molecule, indolactam V was reported to be efficiently convert embryonic stem cells (ESCs) to pancreatic lineage by activating PKC signaling [52]. Similarly, our protocol was efficient in differentiating dental tissue derived MSCs to pancreatic β-cell like cells that secrete insulin upon glucose challenge. During final stage of differentiation, the addition of Exendin-4 and Activin A resulted in the expression of β cell pan-markers. Exendin-4 is a potent GLP-1 agonist that stimulates both β-cell proliferation and neogenesis from ductal progenitor cells [53]. Although all three types of MSCs isolated from human dental tissue showed transdifferentiation potential toward pancreatic lineage, MSCs from follicle tissue had higher propensity to differentiate toward pancreatic lineage when compared with those from pulp and papilla. Therefore, the present study suggests that MSCs from different anatomical regions of dental tissue possess varied differentiation potential toward different lineages despite their identical donor origin.

## Conclusions

Mesenchymal stem cells isolated from dental pulp, papilla, and follicle tissues exhibited largely similar features related to morphology, proliferation, expression of various cell surface, intracellular and pluripotent markers, and differentiation toward mesenchymal lineages such as osteocytes, adipocytes, and chondrocytes. Further, all three types of MSCs displayed greater ability to transdifferentiate into pancreatic β cell-like cells. Nevertheless, differentiated cells were positively stained for dithizone and secreted insulin upon glucose challenge. However, the expression of pancreatic lineage specific markers and insulin secretion assay revealed that follicle-derived MSCs are prominent sources for cell-based diabetic therapy. When compared with other tissue sources of MSCs, the findings of the present study are particularly very interesting when we considered an extracted tooth samples sourced from routine medical waste. To our knowledge, this is the first kind of study in which we compare the pancreatic lineage differentiation potential of MSCs from three different regions of dental tissue from a single donor and identify a potent MSCs source for the generation of pancreatic β cell-like cells.

## Supporting information

**Supplementary Figure 1:** 

## Abbreviations

ADMEM: advanced Dulbecco’s modified Eagle’s media
ADMSC: adipose tissue-derived mesenchymal stem cell
AGCAN: aggrecan
ANOVA: analysis of variance
BMMSC: bone marrow-derived mesenchymal stem cell
BMP2: bone morphogenetic protein 2
CD: cluster differentiation
COL II: collagen II
DM: diabetes mellitus
DMSO: dimethyl sulfoxide
DPBS: Dulbecco’s phosphate buffer saline
DPSC: dental pulp tissue-derived mesenchymal stem cell
ECL: enhanced chemiluminescence
ESC: embryonic stem cell
FABP4: fatty acid binding protein 4
FBS: fetal bovine serum
FITC: fluorescence isothiocyanate
GCG: glucagon
IBMX: isobutylmethylxanthine
iPSC: induced pluripotent stem cell
Krb: Krebs buffer
LPL: lipoprotein lipase
MSC: mesenchymal stem cell
ON: osteonectin
PCR: polymerase chain reaction
PPARγ: peroxisome proliferative activated receptor γ
RT-qPCR: real-time quantitative PCR
RUNX2: Runt-related transcription factor-2
SOX9: SRY-Box 9
SST: somatostatin
UCBMSC: umbilical cord blood-derived mesenchymal stem cell
WJMSC: Wharton’s jelly-derived mesenchymal stem cell
YWHAZ: tyrosine 3-monooxygenase/tryptophan 5-monooxygenase activation protein, zeta polypeptide
