# Supplementary figures and images for: In vitro differentiation of single donor derived human dental mesenchymal stem cells into pancreatic β cell-like cells
Source: Biosci Rep. 2019 May 21;39(5):BSR20182051. doi: 10.1042/BSR20182051 (PMC6527933; doi:10.1042/BSR20182051)

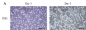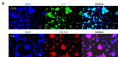

Supplement: Supplementary file 1 [file bsr20182051_Supp1.pdf]
